# Attention deficit hyperactivity disorder: Insights into underfunding in the private healthcare sector in South Africa

**DOI:** 10.4102/sajpsychiatry.v29i0.2050

**Published:** 2023-10-17

**Authors:** Johan J. Botha, Renata Schoeman

**Affiliations:** 1 Department MBA, Faculty of Economic and Management Sciences, University of Stellenbosch Business School, Stellenbosch University, Bellville, South Africa; 2 Department of Leadership, Faculty Healthcare Leadership, University of Stellenbosch Business School, Stellenbosch University, Bellville, South Africa

**Keywords:** attention deficit hyperactivity disorder, ADHD, funding, medical scheme, medical option, comparison, prescribed minimum benefit, benefits

## Abstract

**Background:**

Although the prevalence of attention deficit hyperactivity disorder (ADHD) has remained stable, the number of patients diagnosed with ADHD has increased in recent years owing to increased awareness. Despite this increase, medical schemes in South Africa have not improved their funding models for this condition.

**Aim:**

The study aimed to provide an account of the funding that medical schemes provisioned for treating ADHD in South Africa during 2022.

**Setting:**

All the South African medical schemes that were registered with the Council of Medical Schemes during 2022 (*n* = 72) and all their listed options were evaluated (*n* = 279).

**Methods:**

The study analysed secondary data published on the medical schemes’ websites in the public domain. Statistical minimum, average, maximum and correlation analyses were performed using Excel version 16.58.

**Results:**

Attention deficit hyperactivity disorder is not regarded as a prescribed minimum benefit (PMB) condition and therefore each medical scheme used its own approach to providing its beneficiaries with some or no benefits for ADHD. It was evident that ADHD was underfunded and lacked structured or standardised funding approaches.

**Conclusion:**

Attention deficit hyperactivity disorder is underfunded in the private healthcare sector in South Africa. Better funding models are needed or ADHD needs to be registered as a PMB condition.

**Contribution:**

Findings from this study highlight the urgency for structured and sufficient ADHD-specific funding by medical schemes. Considerations based on these findings may be applied in the National Health Insurance and in other countries around the world.

## Introduction

Attention deficit hyperactivity disorder (ADHD) is being treated as the orphan of mental health disorders in the private healthcare sector in South Africa. This study highlights the lack of proper funding models available for the treatment of this condition in South Africa.

The earliest and most accurate description of ADHD as we know it today came from 1902^1^ when Sir George Still described children with restlessness, inattention and impulsiveness. Attention deficit hyperactivity disorder affects between 2% and 16% of schoolgoing children. The notion that ADHD is only a childhood disorder was discredited in the 1990s with studies demonstrating that ADHD symptoms last well into adulthood.^1,2,3^ Research has revealed that 60% – 70% of children with ADHD continue to have symptoms throughout adulthood.^4^ The prevalence of adult ADHD is between 2.5% and 4.3% of the population.^1,5^

It is well known that the ADHD neurodevelopmental disorder causes patients to suffer from multiple mental health comorbidities, resulting in social, personal and interpersonal challenges negatively affecting the overall quality of life. In addition, patients with ADHD in the South African private healthcare sector have many out-of-pocket (OoP) expenses related to medication and the cost of consultations with a psychiatrist or paediatrician.^6,7,8,9,10,11,12^

A major study concluded that ADHD runs a chronic and costly course, costing governments and patients with ADHD and their families hundreds of thousands of dollars annually.^1^ The costs are divided into direct and indirect costs. Direct costs are the healthcare and OoP expenses that relate to consultations, medication, transportation, food and lodging incurred as a direct result of ADHD healthcare visits.^13^

Indirect costs are those resulting from the time of patients and their families lost because of ADHD, the losses in productivity, the cost of absenteeism and presenteeism, wage losses, the reduction in efficiency and the cost of other disabilities caused by ADHD.^1,14,15^ Some overheads of ADHD are intangible, such as educational and judicial system costs. Educational costs relate to special needs tuition, as required by those with learning disabilities, other health impairments and emotional disturbances. Judicial system costs relate to the costs of larceny, arrests, adjudication, robberies and similar misdemeanours.^16,17^

Many studies conducted across the world during 2019 have determined the annual costs of ADHD per patient. Patients with ADHD had on average $1000 incremental costs per annum in the United States as compared with non-ADHD patients and in Germany the incremental costs were ¨ 2900 per patient per annum.^13,15,18^ A 2017 South African study illustrated that patients were responsible for major OoP expenses relating to the cost of medication, consultations and accessing alternative therapies.^10^ The study further demonstrated that patients with ADHD paid 35.98% of the costs of psychiatric consultations, 41.17% of medicine costs and 62.20% of the costs for supportive therapies. Another finding from this study was that the presence of adult ADHD more than doubled all healthcare-associated expenses of the medical scheme beneficiaries. This study was the largest at the time and included 3 300 000 beneficiaries.^10^ The study calculated the funding availability of the beneficiaries to be only R534 ($62) per beneficiary per annum.

In South Africa, the treatment gap for mental health disorders in the private healthcare sector is 75%. This treatment gap reflects the number of medical scheme beneficiaries (as a percentage) that do not have access to a mental healthcare benefit. The ADHD population in 2017 was between 771 264 (3% of the total population) and 1 285 439 (5% of the total population). This was calculated by multiplying the prevalence (as a percentage) with the population of South Africa during 2017. Using statistical inferential calculations, these figures stand between 1 635 707 and 2 727 168 for 2022.^10^ The prevalence of ADHD and the high OoP expenses created the need to evaluate the current funding structures of the private healthcare sector in South Africa. Never before has the entire medical scheme population of South Africa been evaluated on its funding benefits for ADHD. This article gives an overview of the funding of ADHD for the 72 registered medical schemes and their options during 2022. This is a first for South Africa.^14,19^

Insights into the funding of ADHD indicate where funding can be restructured and improved to match international treatment protocols.

## Study design

This naturalistic study examined the benefits for ADHD management in the private healthcare sector in South Africa during 2022. Secondary data desktop collection and analysis were used as the data were in the public domain. All the medical schemes that were registered under the Council of Medical Schemes (CMS) during 2022 were included.

### Study population and sampling strategy

The study population was the medical schemes (*n* = 72) that were registered in South Africa during 2022. The study sample included all the options of the 72 medical schemes for 2022 (*n* = 279). The total number of beneficiaries belonging to the schemes during 2022 were 8 938 872 members.^19^

### Data collection

Data regarding the availability of specific funds for managing ADHD from each medical scheme were obtained by visiting every scheme’s website domain through a basic online computerised internet search.

### Data analysis

The data collected specifically recorded the open and closed schemes, the four largest scheme administrators, general mental health benefits and ADHD-specific benefits. Each medical scheme and each option of the schemes were assigned a code to make it easier to reference and analyse.

Quantitative data analysis was conducted using Microsoft Excel version 16.58. The data were cleaned by removing the wording in the sentences where the ADHD benefit figures had been stated, leaving only the numbers and figures. The statistical analysis techniques included minimum, maximum, averages and correlation.

## Results

This section includes findings from the administrator analysis, the open and restricted schemes analysis, and a discussion of the general mental healthcare and the ADHD-specific benefits.

### Medical scheme administrators

In South Africa, the 72 medical schemes are run by 26 administrators. The largest four administrators are Discovery, MMI Holdings, Medscheme and Universal Health. These four administrators were compared on ADHD-specific chronic medicine benefits and ADHD benefits in general. As the ADHD-specific chronic medicine benefit was the most common, this was used as comparator. Firstly, an industry correlation analysis was carried out to establish the relationship between the premium paid per single member and secondly, the presence of ADHD chronic medicine benefits were evaluated. The industry correlation coefficient was 0.5, indicating a moderately strong relationship between an increase in the premium paid per member and an increase in ADHD chronic medicine benefits.

### Discovery

At the time of the study, Discovery administered and managed 18 restricted medical schemes on behalf of corporate clients, as well as its own in-house scheme. The total number of beneficiaries managed was 2 800 000 or 31.69% of the South African medical scheme market. Correlation analysis between the premium paid per member and the presence of ADHD-specific chronic medicine benefits revealed a weakly positive relationship for the schemes administered by Discovery (correlation coefficient: 0.3). Moreover, it was found that the Discovery Medical Scheme had no benefits for ADHD across its entire in-house medical scheme. The total number of beneficiaries under Discovery administration that had access to some form of ADHD-specific benefits was 223 845 out of 1 632 306 beneficiaries (treatment gap of 86%). The treatment gap indicates the percentage of beneficiaries in the study population that did not have access to an ADHD-specific benefit (Table 1). Some of the schemes were not included in this analysis because they only had per family per annum chronic medicine benefits (pfpa) and not per member per annum (pmpa) chronic medicine benefits.

**TABLE 1:** Attention deficit hyperactivity disorder chronic medicine benefits of Discovery-administered schemes.

Scheme name	Number of beneficiaries (thousands)	ADHD funds – Chronic meds: pmpa (South African Rand values ’000)	Consultation benefits (South African Rand values ’000)
Anglo	17.65	11.52	0.00
Anglovaal	2.43	0.00	0.00
Bankmed	105.21	25.32	0.00
BMW	3.01	0.00	0.00
Discovery	1339.82	0.00	0.00
Engenmed	3.30	0.00	0.00
LA Health	91.76	7.05	0.00
Libcare	5.83	40.30	12.60
Lonmin	11.41	0.00	0.00
Malcor	4.46	0.00	0.00
Multichoice	3.53	0.00	0.00
Remedi	20.18	0.00	0.00
Retail	12.95	0.00	0.00
TFG	3.17	0.00	0.00
Tsogo Sun	4.20	0.00	0.00
UKZN	3.41	8.83	0.00

ADHD, attention deficit hyperactivity disorder; BMW, Bayerische Motoren Werke AG; TFG, Foschini Group; UKZN, University of KwaZulu-Natal; pmpa, per member per annum.

### Medscheme

Medscheme managed 12 schemes (Table 2), representing 2 900 000 members, at the time of the study. The correlation analysis revealed a moderately strong relationship between an increase in premium paid per member and the presence of ADHD-specific chronic medicine benefits (coefficient: 0.5). Under Medscheme administration, the total number of beneficiaries who had some form of access to ADHD benefits was 1 510 000 (treatment gap of 6%). Only 6% of beneficiaries under Medscheme administration did not have access to a pmpa chronic medicine benefit.

**TABLE 2:** Attention deficit hyperactivity disorder chronic medicine benefits of Medscheme-administered schemes.

Scheme name	Number of beneficiaries (thousands)	ADHD funds – Chronic meds: pmpa (South African Rand values ’000)	Consultation benefits (South African Rand values ’000)
AECI	5.59	0.00	0.00
Barloworld	4.13	0.00	26.18
Bonitas	340.14	12.16	0.00
Fedhealth	71.06	10.30	0.00
GEMS	761.17	11.58	12.38
Horizon	1.40	13.61	13.61
MBMED	4.47	0.00	0.00
Medipos	11.72	4.97	0.00
Medshield	150.09	9.93	21.88
Parmed	2.42	0.00	0.00
SABC	3.93	0.00	0.00
Samwumed	75.55	0.00	0.00
Polmed	174.00	10.18	0.00

ADHD, attention deficit hyperactivity disorder; AECI, AECI Medical Aid; SABC, South African Broadcasting Corporation; GEMS, Government Employees Medical Scheme; MBMED, MBMed Medical Aid Fund; pmpa; per member per annum.

### MMI Holdings

MMI Holdings administered 10 schemes (Table 3) at the time of the study, with a total of 1 600 000 members, and held 18.28% of the market share. The correlation coefficient was 0.4. The total number of beneficiaries under MMI administration who had access to an ADHD benefit was 189 674 (treatment gap of 7%). Only 7% of beneficiaries under MMI administration did not have access to ADHD chronic medicine benefits.

**TABLE 3:** Attention deficit hyperactivity disorder chronic medicine benefits of MMI-administered schemes.

Scheme name	Number of beneficiaries (thousands)	ADHD funds – Chronic meds: pmpa(South African Rand values ’000)	Consultation benefits (South African Rand values ’000)
Bpmas	1.51	7.49	0.00
Engenmed	3.30	0.00	0.00
Fishmed	1.82	0.00	0.00
Golden Arrow	2.44	0.00	0.00
Horizon	1.40	13.61	13.61
Imperial	6.98	23.30	23.30
Momentum	153.26	28.00	28.00
Motohealth	16.95	6.90	6.90
Pick ‘n Pay	7.01	0.00	0.00
Wooltru	9.58	22.73	10.40

ADHD, attention deficit hyperactivity disorder; bpmas, BP Southern Africa Medical Aid Society; pmpa, per member per annum.

### Universal Health

Universal Health administered 11 schemes (Table 4), with 88 494 members, and held 0.99% market share. The correlation coefficient was 0.1 and indicated a very weak positive relationship between an increase in premium paid and an increase in ADHD chronic medicine benefits. The number of beneficiaries who had access to an ADHD benefit under the Universal Health administration was 831 352 (treatment gap of 7%). Only 7% of beneficiaries under Universal Health administration did not have access to an ADHD chronic medicine benefit.

**TABLE 4:** Attention deficit hyperactivity disorder chronic medicine benefits of Universal Health-administered schemes.

Scheme name	Number of beneficiaries (thousands)	ADHD funds – Chronic meds: pmpa (South African Rand values ’000)	Consultation benefits (South African Rand values ’000)
AECI	5.59	0.00	0.00
BCIMA	11.76	0.00	0.00
Compcare	29.40	12.25	0.00
GEMS	761.17	11.58	12.38
Massmart	20.72	0.00	0.00
Omsmaf	30.99	15.35	15.35
Tigerbrands	9.80	8.75	0.00
Transmed	27.97	0.00	0.00
Umvuzo	81.17	0.00	0.00
Witbank Coalfields	24.50	0.00	0.00

ADHD, attention deficit hyperactivity disorder; AECI, AECI Medical Aid; BCIMA, Building and Construction Medical Aid Fund; GEMS, Government Employees Medical Scheme; pmpa, per member per annum.

### Open and restricted schemes

In South Africa, on November 2022, 8 938 872 people belonged to a medical scheme (which is 15% of the total population of 60 600 000). At the time of the study, there were 16 open schemes and 56 restricted schemes in South Africa. The open schemes represented 4 830 000 beneficiaries (54% of the private healthcare population) and the restricted schemes represented 4 110 000 beneficiaries (46% of the total private healthcare population). The number of beneficiaries who had access to some ADHD benefits in the open pool of schemes was 1 700 000 (38% coverage ratio) (Table 5).

**TABLE 5:** Attention deficit hyperactivity disorder chronic medicine benefits from open schemes.

Scheme name	Number of beneficiaries (thousands)	ADHD funds – Chronic meds: pmpa (South African Rand values ’000)	Consultation benefits (South African Rand values ’000)
Bestmed	202.39	10.67	0.00
Bonitas	714.99	12.16	0.00
Cape Medical Plan	8.78	0.00	0.00
Compcare	33.35	12.25	0.00
Discovery	2765.00	0.00	0.00
Fedhealth	148.19	10.30	0.00
Genesis	21.06	0.00	0.00
Health Squared	32.61	4.30	0.00
Keyhealth	67.71	14.85	21.45
Makoti	8.99	0.00	0.00
Medihelp	197.62	12.45	0.00
Medimed	13.87	2.83	28.33
Medshield	154.46	9.93	32.82
Siswe Hosmed	112.80	9.02	19.10
Suremed	2.10	0.00	0.00
Thebemed	25.74	4.00	0.00

ADHD, attention deficit hyperactivity disorder: pmpa, per member per annum.

The number of people who had access to ADHD funds in the restricted pool was 3 340 000 (87% coverage ratio) (Table 6).

**TABLE 6:** Attention deficit hyperactivity disorder chronic medicine benefits from restricted schemes.

Scheme name	Number of beneficiaries (thousands)	ADHD funds – Chronic meds: pmpa (South African Rand values ’000)	Consultation benefits (South African Rand values ’000)
AECI	12.23	0.00	0.00
Alliance-Midmed	3.72	21.91	5.23
Anglo Medical	17.94	11.52	0.00
Anglovaal	4.50	0.00	0.00
Bankmed	219.81	25.32	0.00
Barloworld	11.10	0.00	26.18
BMW	8.05	0.00	0.00
Bpmas	3.40	7.49	0.00
Building and construction	11.76	0.00	0.00
CAMF	48.30	36.60	36.60
De Beers	9.14	41.45	0.00
Engen	6.76	0.00	0.00
Fishmed	4.16	0.00	0.00
Foodmed	18.20	0.00	0.00
Glencore	24.91	0.00	0.00
Golden Arrow	5.26	0.00	0.00
GEMS	1924.57	11.58	12.37
Horizon	6.07	13.61	13.61
Impala	26.58	0.00	0.00
Imperial	16.44	23.30	23.30
LA Health	219.73	23.68	12.60
Libcare	12.93	40.30	12.60
Lonmin	15.12	0.00	0.00
Malcor	11.29	0.00	0.00
Massmart	20.72	0.00	0.00
MBMed	10.20	0.00	0.00
Medipos	25.54	4.97	0.00
Motohealth	39.26	6.90	6.90
Old Mutual	30.99	15.35	15.35
Parmed	4.72	0.00	0.00
PG Group	2.97	0.00	0.00
Pick ’n Pay	15.29	0.00	0.00
Profmed	74.63	17.36	0.00
Randwater	9.01	0.00	0.00
Remedi	47.02	0.00	0.00
Rhodes	21.94	0.00	0.00
Sabmas	24.18	0.00	13.80
SABC	9.49	0.00	0.00
Samwumed	75.55	0.00	0.00
Sasolmed	77.66	25.30	18.99
Sisonke	30.74	5.08	0.00
Polmed	504.76	10.18	0.00
TFG	6.72	0.00	0.00
Tigerbrands	9.79	8.75	0.00
Transmed	31.98	0.00	0.00
Tsogo Sun	11.09	0.00	0.00
Umvuzo	75.72	0.00	0.00
UKZN	6.85	8.82	0.00
Witbank Coalfields	24.65	0.00	0.00
Wooltru	17.99	22.72	10.40

ADHD, attention deficit hyperactivity disorder; AECI, AECI Medical Aid; BCIMA, Building and Construction Medical Aid Fund; CAMAF, Chartered Accountants (SA) Medical Aid Fund; GEMS, Government Employees Medical Scheme; SABC, South African Broadcasting Corporation; TFG, Foschini Group; UKZN, University of KwaZulu-Natal; pmpa, per member per annum.

### General mental health benefits

The most common general mental health benefit was a monetary limit or value that applied to in-hospital benefits, out-of-hospital benefits and chronic medication benefits, or a combination of these. The number of scheme options that included a monetary benefit was 149. This represented 4 800 000 beneficiaries and a coverage ratio of 54%.

The second most common general mental health benefit was the 21-day in-hospital admission benefit (*n* = 102), and 37% of the sample of scheme options (*n* = 279) had this benefit. This amounted to a total of 4 680 000 beneficiaries with a coverage ratio of 52%.

The study found that 20% of scheme options did not have any mental health benefits. This represented 1 400 000 beneficiaries (16%). Furthermore, 20% of medical scheme options only funded prescribed minimum benefit (PMB) conditions. This amounted to 1 380 000 beneficiaries and a coverage ratio of 15%. Also, 31% of medical scheme options had a 21-day drug rehabilitation admission benefit. This represented 3 910 000 beneficiaries and a coverage ratio of 44%. Only 20% of the schemes had the option of forfeiting the 21-day in-hospital admission programme for the benefit of 15 out-of-hospital consultations per annum with either a psychologist or a psychiatrist. This benefit covered 3 870 000 beneficiaries (43% coverage ratio).

The study found that 9% of the medical scheme population had a major depression management and prevention benefit, available to 715 880 beneficiaries (92% treatment gap). A mere 5% of the options had an unlimited telephonic counselling benefit with a psychologist, representing 55 724 beneficiaries (treatment gap of 99%). Also, 5% of options had a 3-day admission benefit for alcohol detoxification, representing 155 038 beneficiaries (2% coverage).

The study found that 9% of medical scheme options had a 3-day admission benefit for proven attempted suicide, available to 2 990 000 beneficiaries (33% coverage). Furthermore, 23% of medical scheme options had a general out-of-hospital consultation benefit with a mental health practitioner, available to 3 810 000 beneficiaries (coverage of 43%).

### Attention deficit hyperactivity disorder-specific benefits

The most common form of ADHD benefit was the registration of ADHD as a treatable condition on the additional disease list (ADL) of schemes or scheme options. This was the case for 7 590 000 beneficiaries (85% coverage ratio). The ADHD was listed as a chronic disease list (CDL) condition on eight of the options, benefitting 137 211 beneficiaries. The ADHD was listed as a non-PMB condition on 10 options, benefitting 134 977 beneficiaries.

The most common ADL benefit was the chronic medicine benefit (7 650 000 beneficiaries), followed by consultation benefits (7 230 000 beneficiaries – 81% coverage ratio) and lastly childhood wellness benefits (1 250 000 beneficiaries). Consultation and childhood benefits are not discussed in this article.

There were 13 options where ADHD benefits were limited to a specific age group, either for children between 2 and 12 years (28 117 beneficiaries), for children between 5 and 18 years (92 048 beneficiaries), for children between 6 and 18 years (138 291 beneficiaries), or for young adults below the age of 21 years (36 181 beneficiaries). There were no options where funding was specifically available for adults with ADHD.

**FIGURE 1:**
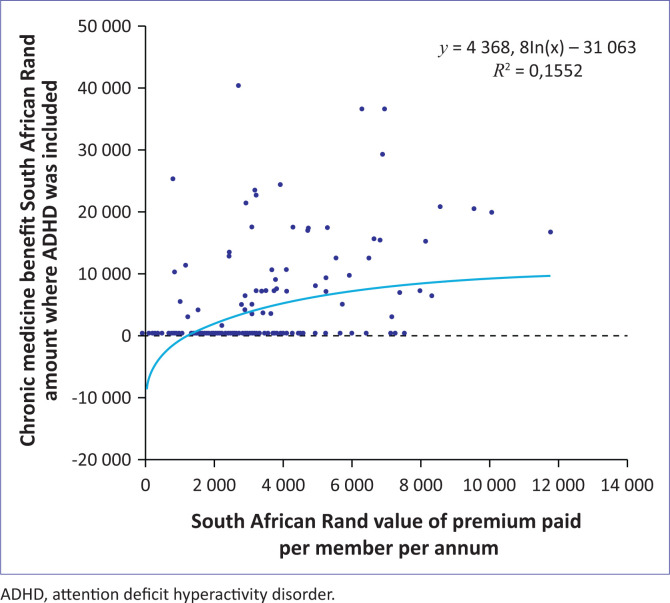
Premium paid in comparison to attention deficit hyperactivity disorder benefits (*N* = 227).

Correlation analysis was performed on the relationship between the presence of ADHD-specific benefits (chronic medicine benefits) and the premium paid per single beneficiary of the medical scheme options. The chronic medicine benefit was chosen for the analysis because it was the most common of all the ADHD-specific benefits (Figure 1).

The correlation coefficient indicates a moderately strong relationship between an increase in premium paid and the presence of ADHD chronic medicine benefits (coefficient: 0.5).

## Key findings

Compared with the open schemes, the restricted pool of schemes had a better ADHD funding coverage ratio. The administrator with the greatest treatment gap for ADHD was Discovery. It is important to observe, however, that an administrator is not responsible for the funding decisions of the individual schemes being administered by it.

There is currently a lack of scientific data regarding administrators of medical schemes in South Africa. Data regarding comparisons between all the medical schemes are also lacking. This is the first comparative study of its kind in South Africa. The study on MMI by Schoeman and De Klerk suggested that funding was insufficient in view of the excessive OoP expenses that patients were required to pay to obtain ADHD management and care.^14^ Further literature revealed, at the respective times of the studies, that 2.5% – 4.3% of adults struggled with prominent ADHD symptoms that impacted their daily functioning significantly,^1,2^ and that none of the medical schemes had adult-specific ADHD benefits. The closest to ADHD-specific benefits were 13 scheme options across all the schemes that dedicated either chronic medicine or consultations under their childhood wellness programmes for ADHD management. These programmes were however limited only to beneficiaries below the age of 21 years.

The funding benefits for ADHD at the time of recent studies were non-specific and non-standardised. According to the South African treatment guidelines for ADHD, a structured protocol is needed in order to manage and treat this condition effectively.^1^ It is clear that current private funding for ADHD falls short when it comes to a structured approach to ADHD management. Of the 279 scheme options, only 66 had a chronic medicine benefit where ADHD was included as an ADL condition. The implications are that these benefits need to be shared among other mental health comorbidities that the patients might have.^13^ This sharing decreases funds available for the treatment of ADHD specifically.

The general mental healthcare benefits indirectly assist people with ADHD who have other mental health comorbidities.^13^ The 21-day in-hospital admission (*n* = 102 medical scheme options) would not assist people with ADHD directly, but indirectly it could help them deal with comorbid conditions such as anxiety, depression or substance abuse, which is rife among ADHD patients.^10^

This study found that 15% of the medical scheme population only had access to treatment and management for PMB conditions. A PMB condition is a disease or ailment for which a medical scheme must pay in full, as required by PMB legislation. In South Africa there are 270 PMB conditions. In essence, a PMB is a defined set of benefits, which ensure that all members of medical schemes have access to minimum health services. The notion that only PMBs are to be covered should be reviewed, because – with the current omission of ADHD as a PMB condition – this policy automatically excludes 1 340 000 members from accessing some form of ADHD benefit. It is recommended that ADHD be registered as a PMB condition requiring structured funding benefits for all age groups affected and policies need to be implemented for this to materialise. There is a need for a cost–benefit study to determine the overall impact of registering ADHD as a PMB condition, as opposed to increasing and standardising ADHD funding benefits across the schemes.

There was a 56% treatment gap for the 21-day in-hospital drug rehabilitation programme, which is concerning because patients with ADHD often have comorbid substance abuse problems.^10^ There was a 92% treatment gap for major depression management programmes, which is problematic owing to the high levels of comorbid depression among ADHD patients.

Previous research showed that the average incremental costs per patient with ADHD were around €2000 per patient with ADHD per annum.^13,15,18^ In rand terms, this amounted to R49 532 on average per patient (using the July 2022 exchange rate). In South Africa, the burden rested on patients to pay OoP expenses of nearly 35.98% of the costs of psychiatric consultations, 41.17% of medication to treat ADHD and 62.20% of the costs of supportive and alternative therapies. This is a further indication of the lack of proper funding models for ADHD in South Africa.

The findings also indicated that ADHD benefits were only available for the more expensive options of the schemes. This implies that only affluent members could benefit from ADHD funds and benefits, whereas the lower income groups would not have any benefits for ADHD.

The main contribution of this study is the evidence that ADHD is only funded as a bundled benefit together with all the other conditions listed on the ADL, non-PMB and non-CDL of medical schemes. The fact that the benefits for ADHD need to be shared with other listed conditions implies that the funding figures are, in fact, lower than found, and this should be taken into account.

## Implications and recommendations

Attention should be paid to the cost savings that would be derived from the better funding of ADHD if implemented by the schemes. Costs related to accidents, absenteeism and presenteeism, as well as the direct and indirect costs related to ADHD, could be greatly reduced if better funding is structured for ADHD. Cost savings will also result from ADHD beneficiaries requiring fewer admissions into mental healthcare institutions, fewer consultations and less drug rehabilitation support if these are better controlled and stabilised.

A discussion between the council of schemes and the administrator that had minimal coverage ratios for ADHD (in this case Discovery) to make them aware of the need for better funding models and more benefits, specifically for ADHD, needs to take place. Even though an administrator is not directly responsible for the funding decisions of the individual schemes it administers, it could influence the schemes to create better funding models.

The overall general mental healthcare package should be optimised to close the treatment gaps. Taking into account the results of this study, the two benefits that need to be improved are the 21-day in-hospital admission and the monetary benefit. The 16% of beneficiaries (1 400 000 people) that do not have any form of mental health benefits need to be reduced to zero. This 16% is considered to be insufficient because mental healthcare-related conditions are on the increase.

It is expensive for patients to consult with psychiatrists. Now that it is clear that patients pay major OoP expenses for psychiatry consultations, owing to the limited funding available for ADHD, it would make sense to upskill the general practitioner population in order to raise the diagnostic threshold of ADHD. General practitioners are the custodians or gatekeepers of any healthcare ecosystem, and if they could become accredited by doing an ADHD diploma, they could be empowered to diagnose and initiate treatment for ADHD patients. This added expertise provided by an ADHD-specific accreditation will not only result in patients saving on psychiatrist consultation fees but will also alleviate pressure on the psychiatrist profession.

The open pool of schemes should be targeted first when better funding models need to be implemented (the open pool had a treatment gap of 62%). The Council for Medical Schemes needs to become more aware and mindful of the need for ADHD to be structurally funded. A structured funding model could include standardised medications and treatment packages that are included across the board in all options of all schemes.

A future study could look at the cost–benefit analysis of either creating specific structured funding models for ADHD as opposed to registering ADHD as a PMB condition with unlimited benefits. A discussion is also recommended regarding the steps needed for ADHD to be registered and recognised as a PMB condition in South Africa. If ADHD could be registered as a PMB condition, this would imply unlimited funding being made available by all medical schemes and would end the debate about requiring better funding models.

Efforts should be established to develop a well-structured clinical care pathway for ADHD in the private sector in South Africa. A clinical pathway will result in cost savings for patients, medical schemes and the government.^20^

In addition, the recognition of ADHD as a PMB condition in South Africa might ensure that patients are eligible for unlimited chronic treatment and other benefits.^21^

## Strengths and limitations

The strength of this study is that it is the first to analyse ADHD benefits across every medical scheme in South Africa, laying the foundation for future ADHD funding-related studies. A limitation was that only the private healthcare sector was evaluated and not the public sector’s management of ADHD. The implication is that if the funding and management of ADHD in the private healthcare sector is scarce, the situation in the public sector could be assumed to be worse owing to a lack of resources and inadequate treatment guidelines.

Other limitations were that there were no exact figures available for the numbers of beneficiaries for every option of every scheme for 2022. The treatment gaps were calculated on exact numbers of beneficiaries obtained from 2021 data. Another limitation is the fact that only public domain data were evaluated; thus, in-house scheme benefits that are available to patients after registration were not taken into account.

## Conclusion

This study gives a first-ever overview of the total funding available for the treatment of ADHD across all the medical schemes in the private healthcare sector during 2022 in South Africa. It is evident that in the time period under investigation, namely 2022, there were no structured or proper funding models for ADHD, as illustrated by the erratic funding for ADHD across the four largest scheme administrators and the individual schemes themselves. The most important finding was that the existing ADHD-specific benefits were insufficient and scantly distributed across the scheme population.

Attention deficit hyperactivity disorder, as a recognised mental disorder, needs proper protocols and sufficient funding. This article provided evidence that ADHD is underfunded and that clear, well-structured funding benefits and models are recommended.
